# The optimal system of care for the management of delayed sleep onset in adult ADHD in the UK: a modified Delphi consensus

**DOI:** 10.3389/fpsyt.2025.1566390

**Published:** 2025-08-14

**Authors:** Philip Asherson, Giovanni Giaroli, Paul Gringras, Heidi Phillips, Hugh Selsick, Michael Smith, Dietmar Hank

**Affiliations:** ^1^ Social, Genetic and Developmental Psychiatry Centre, Institute of Psychiatry, Psychology and Neuroscience, King's College, London, United Kingdom; ^2^ The Giroli Centre, London, United Kingdom; ^3^ Paediatric Sleep Department, Evelina Children's Hospital, King's College London and Guy's and St Thomas' NHS Foundation Trust, London, United Kingdom; ^4^ Swansea University Medical School, Swansea, United Kingdom; ^5^ Insomnia and Behavioural Sleep Medicine Clinic, University College London Hospitals, London, United Kingdom; ^6^ Leeds NHS ADHD Service, Leeds and York Partnership NHS Foundation Trust, Leeds, United Kingdom; ^7^ Adult ADHD Service, Avon & Wiltshire Mental Health Partnership Trust, Bath, United Kingdom

**Keywords:** attention deficit hyperactivity disorder, attention deficit disorder with hyperactivity, delayed sleep onset, Delphi consensus, UK

## Abstract

**Introduction:**

Sleep-related disorders affect a significant number of individuals with ADHD, the most common of which has been found to be delayed sleep phase syndrome/delayed sleep onset. The presence of a sleep disorder can exacerbate ADHD symptoms and impair cognitive functions. Despite the significance of these issues, they are often overlooked, potentially leading to unsafe self-medication practices and illicit substance abuse. While the literature supports the efficacy of melatonin in treating delayed sleep onset among children and adolescents with ADHD, evidence in adults is less well-established. This consensus study aims to establish consensus among healthcare professionals regarding the overall management of adults with ADHD experiencing delayed sleep onset in the UK, with the aim of guiding good clinical practice.

**Methods:**

The process employed a modified Delphi methodology. A literature review was conducted to understand the current evidence base. A steering group of seven experts from the UK attended a virtual meeting in April 2024. During this meeting, facilitated by an independent moderator, the group identified six primary domains. Based on these domains, 40 statements were developed into an online survey for testing with a wider panel of peers.

Stopping criteria for consensus rounds were established as a survey duration of four months, a target of 200 responses, and the requirement that at least 90% of the statements achieve the consensus threshold of ≥75% agreement.

**Results:**

A total of 212 responses were received from healthcare professionals experienced in managing adult patients with ADHD and sleep disorders in the UK. All proposed statements achieved consensus, with 90% of statements achieved ≥90% agreement (n=36/40).

**Conclusion:**

Based on the agreement levels achieved, the steering group developed a series of recommendations for the management of delayed sleep onset in adult ADHD in the UK. Given the prevalence of sleep disorders in this population, a comprehensive management approach is essential. This should include effective treatments, such as melatonin, which could be initiated in primary care and monitored by general practitioners for newly diagnosed patients. It is also important that treatment for delayed sleep onset is continued as individuals transition from childhood into adulthood.

## Introduction

Attention deficit hyperactivity disorder (ADHD) is characterised by inattention and/or hyperactivity and impulsivity, and negatively impacts quality of life (1, 2). Several studies have reported a significant increase in mortality and unintentional injuries leading to emergency department visits or hospitalisations among untreated patients with ADHD (3–5). Historically, ADHD was viewed as a diagnosis limited to childhood and adolescence with the assumption that it resolves by adulthood. However, it is now recognised that ADHD can persist into adulthood in 40–60% of cases (6, 7).

Despite the National Institute for Health and Care Excellence (NICE) estimating the prevalence of ADHD among UK adults to be approximately 3–4%, and a recent increase in referrals for assessment and adult diagnoses, the disorder remains significantly underdiagnosed (2). Furthermore, there are considerable variations in the time to diagnosis for adults across the country, ranging from 12 weeks to over 10 years, with some regions no longer able to provide support (8). The management of ADHD is now recognised as a major healthcare concern in the UK, in part because of the association of ADHD with a wide range of mental and physical health comorbidities (9), including the frequent co-occurrence of sleep disorders (10).

Sleep-related disorders affect a significant number of individuals with ADHD (11, 12), and the most common have been found to be delayed sleep phase syndrome (DSPS; 36% of individuals), insomnia (30.6%), and restless legs syndrome (RLS)/periodic limb movement disorder (PLMD; 29%) (11). Both ADHD and sleep disorders have been shown to significantly impact daily functioning and cognitive performance (13), and the severity of ADHD symptoms is greater in individuals with co-occurring sleep dysfunction (14).

ADHD symptoms may contribute to prolonged sleep onset latency and a decline in sleep quality. A 2010 study (12) found that up to 78% of individuals with ADHD are affected by delayed sleep onset, with dim-light melatonin onset (DLMO) being delayed by an average of 1.5 hours. This circadian rhythm disorder can be improved through the use of both non-pharmacological and pharmacological approaches.

The mechanism by which ADHD contributes to sleep dysfunction is complex, but it has been suggested that several factors may contribute, including:

- Lower than normal dopamine D_2_/D_3_ receptor availability in the hypothalamic region of ADHD (15)
- Norepinephrine and dopamine dysregulation which contributes to ADHD symptoms and arousal regulation (16)
- Core deficits in in self-regulation, including planning and sleep hygiene (17)

Despite the high rate of sleep disorders in ADHD, healthcare professionals may struggle to complete sleep assessments due to limited time, the presence of co-morbid conditions, and insufficient training in sleep medicine (18). Medical interventions may include the inappropriate use of sedative antidepressants and hypnotics regardless of the cause of the sleep problem. In addition, many individuals with ADHD, in an effort to improve sleep, often resort to self-medication strategies, including the use of over-the-counter hypnotics, antihistamines, diphenhydramine products, pain relief medications, herbal remedies, vitamins, minerals, and illicit substances such as cannabis, as well as alterations to diet and exercise, all of which may pose health risks (19, 20).

One medication used to treat sleep problems in ADHD is Melatonin, a hormone used to treat some sleep disorders due to its effectiveness in normalising the sleep-wake cycle (21). In the UK melatonin is approved for prescription use for treating insomnia in children and adolescents with ADHD, Autism Spectrum Disorder, and specific neurogenetic disorders that present with aberrant diurnal melatonin secretion, as well as for nocturnal awakenings and primary insomnia in adults age 55 and older (22–24). Specifically, there is a dearth of randomised controlled trial evidence specific to this population, with only one such study found (25). However, several opinion statements and other publications support the use of melatonin for sleep improvement (25–28). Due to the limited evidence, melatonin is not approved in the UK for use in most adults with ADHD and is not available over the counter as it is in other countries. Therefore, a current unmet need exists in the UK regarding the appropriate use of melatonin in the management of adults with ADHD who experience delayed sleep onset [including Delayed Sleep Phase Syndrome (DSPS)], likely due to a limited awareness of circadian rhythm disorders among general practitioners and limited research evidence on the use of melatonin in this population (22).

This modified Delphi consensus study aimed to establish consensus among healthcare professionals regarding the overall management of adults with ADHD experiencing delayed sleep onset in the UK, with the aim of guiding good clinical practice.

## Methods

**Figure 1:**
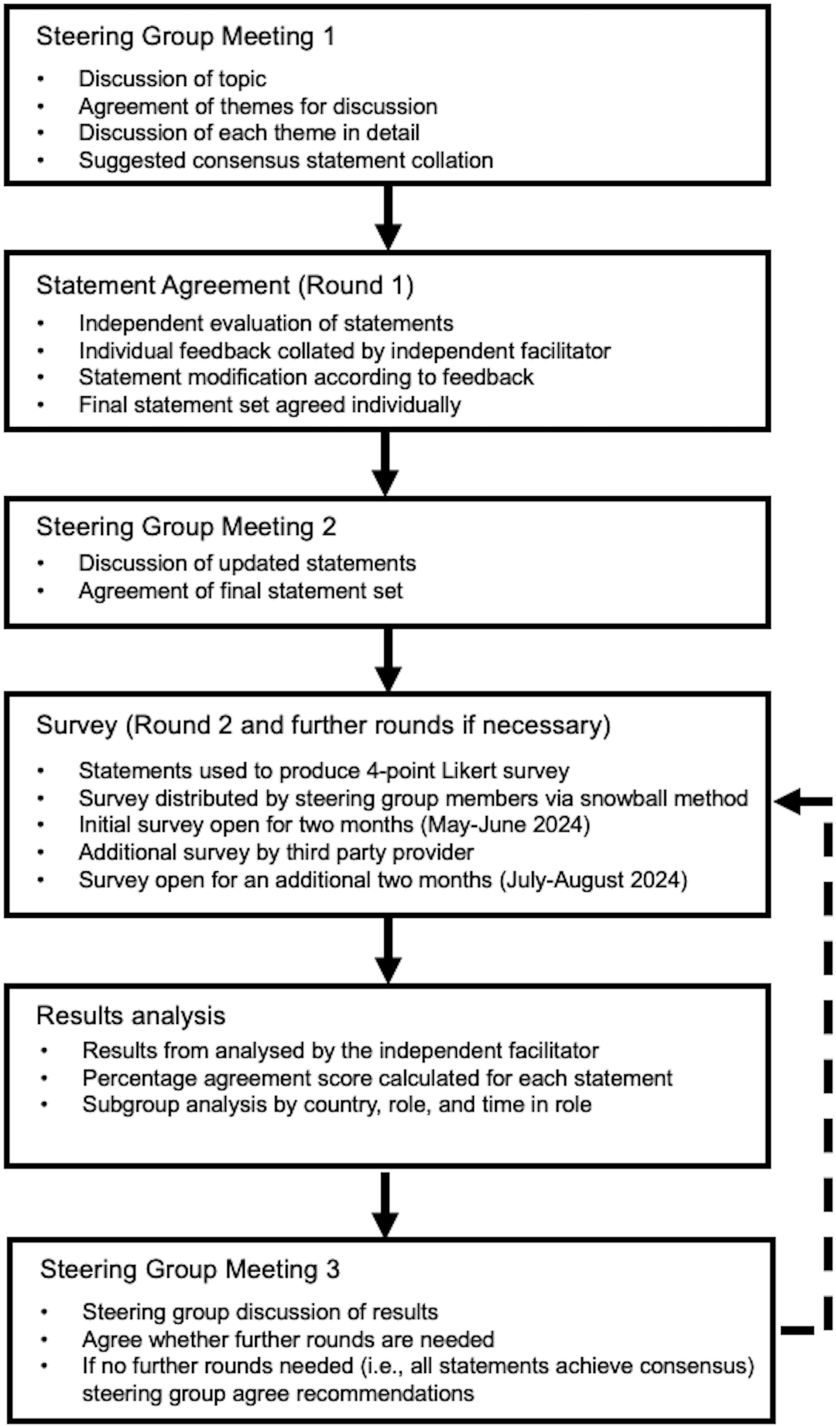
Modified Delphi study design.

The process employed a modified Delphi methodology (**Figure 1**). This method was chosen to gather expert opinions using established methods, as it is recognised as a reliable tool (29).

In March 2024, a brief literature review, performed by the independent facilitator (Triducive Partners Ltd, a healthcare agency specialised in the delivery of Delphi consensus) was carried out to understand the current evidence base and shape the agenda of a steering group meeting (see **Supplementary Data**; **Supplementary Figure S1**).

The steering group included seven UK healthcare professionals with expertise in either ADHD, sleep disorders, or both, who were invited to participate in the study via email. The group convened in April 2024 to discuss the current challenges and opportunities in the management of delayed sleep onset in adults with ADHD in the UK. The selection of the steering group was based on professional roles, experience, and publication history and included three Consultant Psychiatrists, one Sleep and Neurodisability Consultant, one Specialist GP in Neurodiversity, one Professor of Neurodevelopmental Psychiatry, and one Medical Director. During the initial meeting, and facilitated by the independent facilitator, the group discussed the current barriers and opportunities in the management of delayed sleep onset in adults with ADHD. After discussion, the key points were agreed and grouped into six logical domains of focus for the consensus, these were:

A. The burden of delayed sleep onset in adult patients with ADHD
(To understand the levels of recognition of the burden amongst respondents)
B. The need to assess delayed sleep onset in adult patients with ADHD
(To understand attitudes towards assessment of sleep in individuals with ADHD)
C. Principles of delivering treatment and care for delayed sleep onset in adult patients with ADHD in the UK
(To examine those aspects of service delivery that may improve management)
D. Optimising management of delayed sleep onset in adult patients with ADHD
(To establish agreement over what processes need to be in place to deliver optimal care)
E. Policy and guideline requirements
(To establish principles of policy and guidelines that should be adopted into UK practice)
F. Additional evidence, education, and training support

(To gain support for patient and HCP education and materials targeted at delayed sleep onset in ADHD)

The steering group collaboratively discussed each domain and for each one formed a set of consensus statements. These statements were then collated and evaluated independently by the group members, who rated them as either “accept”, “remove”, or “reword with suggested changes”. Recommendations were accepted based on a simple majority. This process resulted in the initial round of consensus with 40 statements agreed by the group for further testing.

The agreed statements were developed into a four-point *Likert* scale survey (“strongly agree”, “tend to agree”, “tend to disagree”, and “strongly disagree”). The 4-point Likert scale chosen to encourage respondents to think about their agreement vs disagreement rather than simply opting for a neutral middle ground. This survey was distributed by the steering group using a snowball sampling method over a two-month period from May to June 2024. Additionally, a third party specialised in market research (M3 Global) facilitated the collection of further responses in July and August 2024. M3 Global holds a large panel of healthcare professionals which they recruited respondents from according to the following criteria:

- Current practice as either general practitioner (GP) specialising in mental health or sleep disorders, mental health service specialist (MHSS), or sleep disorder specialist (SDS)
- Located within the UK (England, Scotland, Wales, and Northern Ireland)

Anonymity of responders was planned into the study design; thus, the identity of respondents was not disclosed to the steering group or the independent facilitator. No personal information beyond demographic data (current role, time in the current role, and country) was captured during the survey. All collected responses were incorporated into the final analysis.

Stopping criteria were agreed as a four-month survey window, a target of 200 responses, and a requirement that 90% of statements meet the consensus threshold set at 75% (a commonly used standard) (30). These criteria were designed to ensure an adequate response rate while considering the time constraints inherent in the healthcare system.

A statement of consent was included at the start of the survey and consent was provided by each respondent prior to participation. Since this study only collected the anonymous opinions of healthcare professionals and no patient-specific data was captured, ethical approval was not sought.

Completed surveys were analysed to produce an overall agreement level (i.e., the number of respondents expressing agreement as a percentage of the overall number of respondents for each statement). The results were subsequently reviewed by the members of the steering group to identify potential recommendations and conclusions based on the responses received. The analysis of Round 2 was carried out in September 2024, and the steering group reconvened on 7^th^ October 2024 to discuss and evaluate the results.

To present the findings of the current study, ACCORD (ACcurate COnsensus Reporting Document) guidelines were adhered to (31).

### Patient and public involvement

There was no patient or public involvement in this study. The primary objective was to assess the perspectives of healthcare professionals regarding the optimal care system for the management of delayed sleep onset in adults with ADHD in the UK.

## Results

The results are presented in the descriptions below. Statements (see **Table 1**) are referred to by domain and sequential number, (e.g., Statement 1 = A1, Statement 9 = B9, etc.).

**Table 1:** Defined consensus statements and corresponding levels of agreement (all numbers rounded to the nearest whole number).

No:	Statement	Strongly Agree	Tenda To Agree	Tend To Disagree	Strongly Disagree		Agreement
Domain A: The burden of delayed sleep onset in adult patients with ADHD							
A1	The burden of delayed sleep onset in adult patients with ADHD should always be recognised	63%	35%	1%		1%	98%
A2	Adult patients with ADHD, their families and social networks can be negatively affected by delayed sleep onset	67%	28%	3%		1%	96%
A3	Delayed sleep onset exacerbates the ADHD symptoms of inattention, hyperactivity, and impulsivity	60%	36%	2%		1%	96%
A4	Delayed sleep onset aggravates functional impairments, including social activities, personal relationships, education, and employment	63%	33%	2%		1%	97%
A5	Delayed sleep onset contributes to worsening of co-occurring low mood, depression, emotional dysregulation, daytime sleepiness, and fatigue	69%	26%	4%		1%	95%
A6	Delayed sleep onset contributes to poor health outcomes associated with ADHD, such as increased risk of smoking, obesity, type II diabetes, and metabolic issues	47%	42%	9%		2%	89%
A7	Untreated delayed sleep onset may lead to patients seeking self-medication strategies	58%	37%	2%		2%	96%
A8	Management of delayed sleep onset should be integral to a comprehensive approach for adult patients with ADHD	64%	34%	1%		1%	98%
Domain B: The need to assess delayed sleep onset in adult patients with ADHD							
B9	Delayed sleep onset is the most common sleep problem in adult patients with ADHD	23%	54%	19%		3%	77%
B10	For every diagnosed adult patient with ADHD, their sleep pattern and quality should ideally be reviewed at every appointment	49%	45%	6%		0%	94%
B11	Healthcare professionals should seek to evaluate poor sleep hygiene practices that might exacerbate delayed sleep onset in adult patients with ADHD	65%	32%	3%		0%	97%
B12	Healthcare professionals should consider other types of sleep problems (e.g., restless legs syndrome (RLS), sleep apnoea, insomnia) when determining the cause of delayed sleep wake phase disorder in adult patients with ADHD	63%	35%	1%		1%	98%
B13	Healthcare professionals should evaluate the impact of delayed sleep onset on adult patients with ADHD	67%	31%	1%		1%	98%
Domain C: Principles of delivering treatment and care for delayed sleep onset in adult patients with ADHD in the UK							
C14	Optimising ADHD treatment may help improve symptoms associated with delayed sleep onset	51%	46%	2%		0%	97%
C15	Enhancing collaborative (transition) arrangements between child and adult ADHD services could improve outcomes for adult patients with ADHD and delayed sleep onset	53%	43%	4%		0%	96%
C16	Effective, prescribed medication, including for the treatment of delayed sleep onset and ADHD, should be continued when patients transition to adult ADHD services	56%	40%	4%		0%	96%
C17	Enhancing collaborative (transition) arrangements between primary care and adult ADHD services could improve outcomes for adult patients with ADHD and delayed sleep onset	53%	43%	3%		0%	97%
C18	Non-pharmacological therapies for delayed sleep onset should always be considered prior to the use of prescribed medications	64%	31%	5%		0%	95%
C19	Psychoeducation about delayed sleep onset is essential in optimising outcomes for adult patients with ADHD and delayed sleep onset	70%	29%	0%		0%	99%
C20	Special attention should be given to the effect of stimulant dose timing and formulation on sleep problems	67%	31%	1%		1%	98%
C21	Stimulants can improve delayed sleep onset in some cases, and worsen delayed sleep onset in others	37%	55%	8%		0%	92%
Domain D: Optimising management of delayed sleep onset in adult patients with ADHD							
D22	The ongoing use of baseline questionnaires, such as a sleep diary for at least 7 days, to monitor delayed sleep onset will help optimise patient management	35%	55%	10%		0%	90%
D23	In addition to a medical history and sleep diary, data on sleep timing and patterns from personal monitors can help support a diagnosis of delayed sleep onset	40%	53%	7%		1%	92%
D24	Adult patients with ADHD and delayed sleep onset should not face a lack of access to effective treatment options	69%	30%	1%		0%	99%
D25	Where other interventions have not succeeded in addressing delayed sleep wake phase disorder, healthcare professionals should have the ability to initiate the use of melatonin for patients under 25 years old, knowing that treatment can be continued in primary care	52%	39%	9%		0%	91%
Domain E: Policy and guideline requirements							
E26	All neurologists with an interest in sleep medicine should be able to manage delayed sleep onset in adult patients with ADHD	45%	46%	8%		1%	91%
E27	All adult mental health services, including community mental health teams, non-medical prescribers, as well as GPs, should be able to manage delayed sleep onset in adult patients with ADHD	45%	39%	14%		2%	84%
E28	Policy makers should recognise that when effective medications cannot be prescribed, patients may resort to self-medication, including the harmful use of alcohol, cannabis, and illegally sourced prescription medications	63%	31%	4%		1%	94%
E29	The use of medicines should be based on clinical need rather than cost considerations	57%	33%	8%		1%	90%
E30	Healthcare professionals confident in managing sleep disorders should be able to prescribe melatonin, when appropriate, for delayed sleep wake phase disorder for patients under 25 years old, regardless of whether they are in primary or secondary care	57%	35%	6%		2%	92%
E31	Melatonin for delayed sleep wake phase disorder for patients under 25 years old should be permitted to be initiated and prescribed in primary care	48%	36%	12%		4%	84%
Domain F: Additional evidence, education, and training support							
F32	In some cases, implementing general sleep hygiene practices could improve delayed sleep onset in adult patients with ADHD	58%	40%	2%		0%	97%
F33	GPs require the best resources/materials about general sleep hygiene education to pass on to patients with ADHD	67%	30%	2%		0%	98%
F34	Healthcare professionals should preferably receive education and training regarding the diagnosis and management of delayed sleep onset in adult patients with ADHD	67%	31%	2%		0%	98%
F35	Families and relatives should be educated that the condition may be a biologically informed/driven problem, that might be beyond the patient's control	62%	34%	3%		0%	96%
F36	All adult patients with ADHD should be provided with explanations for delayed onset of sleep	67%	30%	3%		1%	96%
F37	Adult patients with ADHD should be advised and supported with behavioural strategies to minimise ADHD-related impairments	71%	28%	1%		0%	99%
F38	Healthcare professionals should be aware of all available treatment options for delayed sleep onset in adult patients with ADHD	69%	27%	2%		1%	97%
F39	Proper shared protocols between primary care professionals and specialists should be developed	72%	26%	1%		0%	99%
F40	Prescribing guidelines should be developed as flowcharts to ensure easy understanding and compliance by healthcare professionals	72%	26%	1%		0%	98%

### Round 1

**Figure 2:**
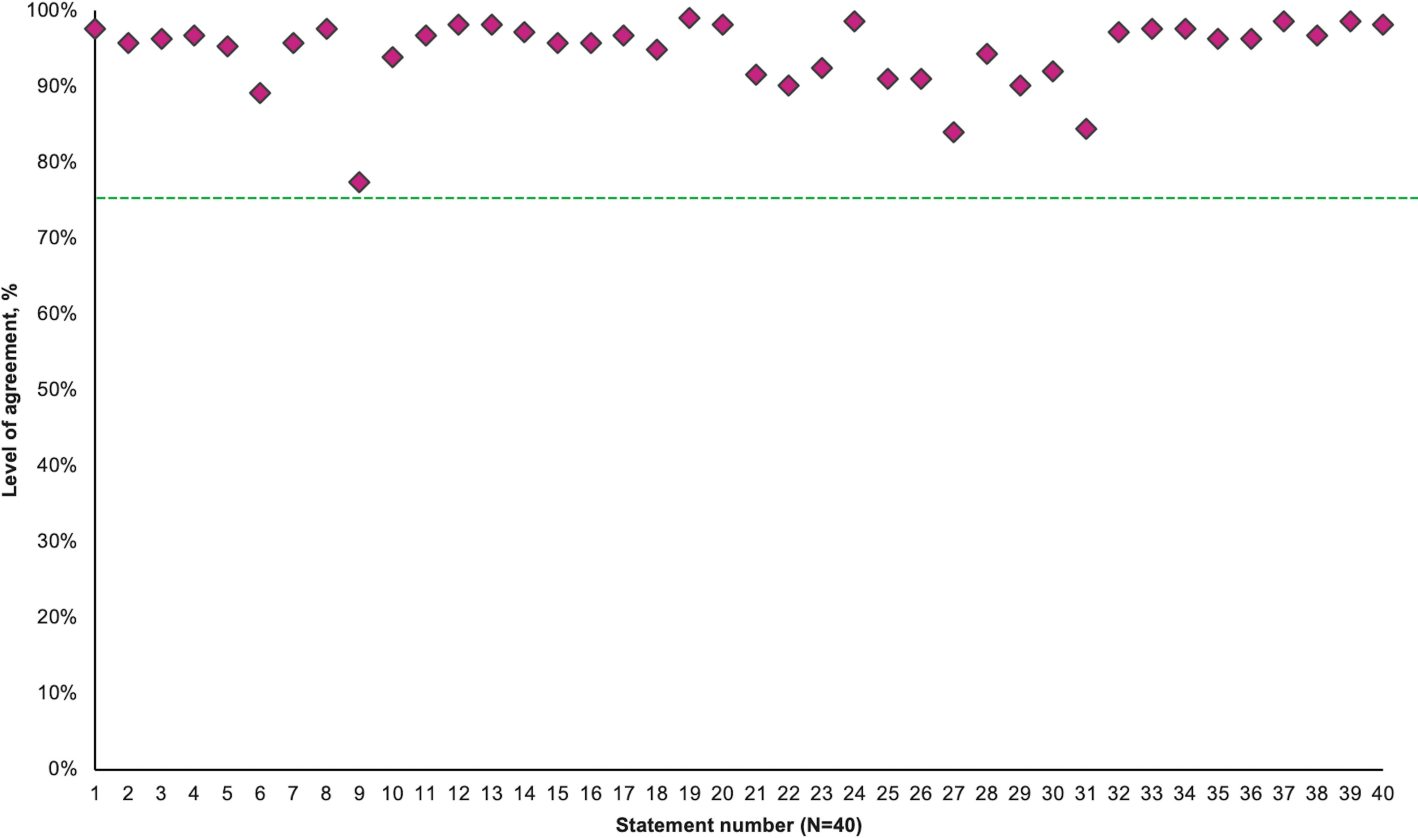
Consensus agreement levels by statement. The threshold for consensus is depicted by the green line (75%).

During the first round of statement testing with the members of the steering group, a final set of 40 statements was considered appropriate for testing in round 2 with the wider survey panel (**Table 1**; **Figure 2**).

### Round 2

Completed surveys were received from 212 practitioners with experience in the management of adult patients with ADHD and/or delayed sleep onset. All responses were included in the final analysis. Respondent role, time in role and country are shown in **Supplementary Figures S2**–**S4**.

Consensus was achieved for all statements during Round 2 (**Table 1**; **Figure 2**). Distribution of consensus scores on the four-point *Likert* scale provided to respondents is represented in **Supplementary Figure S5**. This distribution shows some variation in the strength of agreement between statements that have achieved consensus, specifically statements 9, 21,22, 23, and 26 exhibited a greater proportion of ‘Tend to agree’ responses than ‘Strongly agree’ so these may be considered to be less strongly supported by respondents.

#### Domain A: the burden of delayed sleep onset in adult patients with ADHD

There is a high level of agreement regarding the statements in Domain A. Nevertheless, these statements are rarely discussed in detail in real-world settings, despite healthcare professionals recognising their significance (A3, 96%; A4, 97%; and A5, 95%).

Overall, there appears to be a disconnect between understanding of the burden of delayed sleep onset in ADHD and the practical implementation of strategies described in this section. Respondents agreed that the management of delayed sleep onset should be included in a comprehensive care strategy for adult patients with ADHD (A8, 98%). It is important for healthcare professionals to consistently recognize the burden of delayed sleep onset in adult ADHD patients (S1, 98%), as this issue can impact not only the patients themselves but also their families and social networks (A2, 96% and A4, 97%).

#### Domain B: the need to assess delayed sleep onset in adult patients with ADHD

All statements in this domain achieved a high level of consensus. However, agreement was lower for B7 (77%), which may be attributed to a lack of familiarity with the specific term “delayed sleep onset.”

#### Domain C: principles of delivering treatment and care for delayed sleep onset in adult patients with ADHD in the UK

Respondents agreed that optimising ADHD treatment plays a key role in alleviating symptoms of delayed sleep onset (C14, 97%). Improved collaboration between child and adult ADHD services, as well as between primary care and adult ADHD services, could significantly improve outcomes for adults with ADHD who experience delayed sleep onset (C15, 96%).

There was also agreement on the importance of psychoeducation, and that non-pharmacological therapies should be prioritized before medication (C18, 95%; S19, 99%). It is also important that continuity of prescribed medications for ADHD and delayed-sleep onset is in place as young people transition to adult ADHD services (C16, 96%).

The timing and formulation of stimulants, the most common medication prescribed in ADHD, requires careful titration to individual patient responses, as respondents agreed that this can impact sleep onset (C20, 98%; S21, 92%).

#### Domain D: optimising management of delayed sleep onset in adult patients with ADHD

Using baseline questionnaires, such as a 7-day sleep diary, to monitor delayed sleep onset can improve patient management (D22, 90%). In conjunction with traditional paper diaries, a variety of online sleep diaries are available, serving as essential tools for the diagnosis, behavioural treatment, and monitoring in patients with sleep disorders. Furthermore, in addition to medical history and sleep diary data, personal monitors that track sleep timing and patterns can aid in the diagnosis of circadian rhythm disorders (D23, 92%).

Respondents also agreed that adults with ADHD and delayed sleep onset should have access to effective pharmacological treatment to alleviate symptoms associated with sleep disorder (D24, 99%). There was agreement that where non-pharmacological treatments for circadian rhythm disorder are ineffective, healthcare providers should consider prescribing melatonin, with ongoing management in primary care (D25, 91%).

#### Domain E: policy and guideline requirements

All statements in this domain achieved consensus. E27 and E31 both achieved consensus with 84% agreement, supporting the involvement of general practitioners in initiating treatment within the primary care setting.

There was strong agreement that restricted access to effective medications may prompt certain patients to self-medicate (E28, 94%). Consequently, the prescription of properly licensed sleep medications by qualified healthcare providers would be advantageous (E30, 92%).

#### Domain F: additional evidence, education, and training support

All statements in this domain achieved a high level of agreement, underscoring the necessity of providing education for healthcare professionals in this area. Respondents recognised the need for healthcare professionals to receive comprehensive training in both the diagnosing and management of the condition (F34, 98%). Furthermore education of patients and their carers on effective sleep hygiene practices and behavioural support strategies was agreed (F32, 36, and 37, 97%, 96%, and 99%, respectively). Clinicians should be aware of all available treatment options for addressing delayed sleep onset in adults with ADHD (F38, 97%).

## Recommendations

Based on these results, the steering group recommend the following strategies to optimise the system of care for adults with ADHD-associated delayed sleep onset in the UK:

- Given the high prevalence of ADHD among adults, it is crucial to acknowledge and address delayed sleep onset as a fundamental aspect of ADHD management.
- Healthcare professionals should be knowledgeable about delayed sleep onset related to ADHD and be informed about the available treatment options.
- Adult patients with ADHD should have access to effective treatment options for delayed sleep onset, including both non-pharmacological and pharmacological interventions such as melatonin.
- Melatonin treatment may be initiated in primary care, with patient monitoring by general practitioners.
- For those treated with melatonin in childhood or adolescence, it is important to continue this treatment during the transition to adult care to ensure consistent management of delayed sleep onset and ADHD.
- There is a need to develop prescribing guidelines to improve care continuity and initiation for adult patients with ADHD, delayed sleep onset.

## Discussion

Each recommendation is discussed in this section separately.

### Given the high prevalence of ADHD among adults, it is crucial to acknowledge and address delayed sleep onset as a fundamental aspect of ADHD management

Individuals with ADHD face not only an increased risk of sleep disorder (including circadian rhythm disorders such as delayed sleep onset) (25) but also a 14-fold increased likelihood of requiring a prescribed medication. The prevalence rates for a sleep disorder diagnosis or any sleep medication prescription are highest in middle-aged and older adults with ADHD (32).

ADHD is strongly associated with co-occurrence of delayed sleep onset (25), which negatively impact patients’ quality of life, contribute to additional comorbidities, and exacerbate ADHD symptoms (33). Previous studies have highlighted the association between ADHD and various co-morbid conditions, identifying ADHD as a risk factor for cardiovascular, gastrointestinal, psychiatric, and neurological disorders (9, 10). Chronic poor sleep not only worsens these conditions but is also associated with an increased risk of mortality and can adversely affect painful musculoskeletal and metabolic disorders (34–37). In addition, individuals often experience diminished mental health, cognitive impairments, reduced productivity, and a higher incidence of accidents, unintentional injuries, and suicide. This evidence supports the need to both assess and address ADHD symptoms and sleep disorders in affected pateints (38, 39).

### Healthcare professionals should be knowledgeable about delayed sleep onset related to ADHD and be informed about the available treatment options

Given the high prevalence of ADHD among adults, it is essential for healthcare professionals, particularly those who may not specialise in ADHD, to improve their understanding of the connection between mental and physical comorbidities (40).

Healthcare professionals are encouraged to proactively inquire about sleep patterns (41), and this is particularly important for adults with ADHD, given the high prevalence of the comorbidity (13). Despite evidence indicating that sleep problems are prevalent among ADHD patients globally, healthcare professionals infrequently address issues related to sleep quality and duration (41, 42). Research has identified several factors contributing to this oversight, including time constraints during primary care appointments, competing priorities, and insufficient training on sleep disorders (42).

Expanding knowledge, promoting education, and raising awareness among healthcare professionals regarding delayed sleep in ADHD is an essential component of improving care for adults with ADHD. Insufficient training may result in under-recognition or misspecification of both ADHD and associated comorbidities, thereby hindering effective management (1, 43). As optimal management of ADHD is among the first steps in managing co-occurring delayed sleep onset insomnia, practitioners need to develop skills in the management of both conditions. Training is now becoming important for primary care professionals in the UK, as they are playing an increasingly significant role in the ongoing monitoring of patients with ADHD.

### Adult patients with ADHD should have access to effective treatment options for delayed sleep onset, including both non-pharmacological and pharmacological interventions such as melatonin

Our results support the use of non-pharmacological measures prior to the use of prescription medicines, but the treatment approach should be tailored to the individual. To support appropriate prescribing, it is important to consider all factors that may contribute to delayed sleep onset, including optimising stimulant treatments for ADHD symptoms, and addressing sleep hygiene practices. The expert group held the view that optimising ADHD treatment alone can improve delayed sleep onset in 50% or more of adults with ADHD.

However, if there is an underlying delay in endogenous melatonin production then exogenous melatonin has demonstrated efficacy in advancing the circadian rhythm and reducing ADHD synptoms (25). In practice, this may be determined by either measurement of melatonin levels in saliva (44) or through a validated instrument such as the Munich Chronotype Questionnaire, but these are not generally used in primary care settings (45).

Despite evidence supporting the use of melatonin for delayed sleep onset in children with ADHD (46, 47), there is limited evidence specific to adults with ADHD, and further research is required. In the only randomised controlled trial to date, Van Andel at al (25) found that melatonin alone advanced the DLMO by approximately 1 hour and 28 minutes (p=0.001), while melatonin plus BLT advanced DLMO by about 1 hour and 58 minutes (p<0.001). In addition, the melatonin-only group experienced a reduction in self-reported ADHD symptoms directly after treatment. Despite the limited trial data in adults, the consensus reported here supports the use of melatonin for delayed sleep onset in adults with ADHD.

The use of melatonin may also address the inappropriate use of other medications for sleep problems in ADHD. In the UK, z-drugs are primarily prescribed for sleep-related issues, but recent NHS guidelines have led to approximately a 10% reduction in prescriptions since 2019 due to concerns about dependence and withdrawal effects (48, 49). The steering group also reported the widespread prescribing of sedative antipsychotics and antidepressants in their personal experience, as well as the use of cannabis by patients with ADHD and delayed sleep onset.

In summary, research indicates that melatonin may be effective in addressing delayed sleep onset in adults with ADHD, but use should always be supported by sleep hygiene advise and psychological interventions. Melatonin has a chronobiotic effect that influences the biological clock; therefore, precise timing and dosage are crucial for its use, as incorrect use may exacerbate sleep issues (50). As for all prescribed drugs, it is important to inform patients about potential adverse events and to routinely monitor response to treatment (25–28, 33, 51).

### Melatonin treatment may be initiated in primary care, with patient monitoring by general practitioners

The consensus supported the view that improving sleep hygiene using non-pharmacological therapies combined with psychoeducation should be prioritised initially (52). Non-pharmacological interventions may also include chronotherapy (e.g., phase delay method), bright light therapy to help advance the circadian phase, and behavioural strategies such as cognitive behavioural therapy (53, 54). If these measures prove ineffective, the addition of pharmacological interventions such as melatonin should be considered for adults with ADHD and delayed sleep onset.

Currently, Adult ADHD healthcare services in the UK are facing significant challenges, including escalating demand and unprecedented waiting times, which can, in some instances, exceed 10 years (8, 39). Service provision remains fragmented, with a growing number of private healthcare providers, contributing to inconsistent treatment approaches and patient management (39). Related to this, in the UK many individuals with ADHD remain untreated due to the lack of specialist services. As a result of these problems in service delivery, new models of care for ADHD are being developed and implemented, including a greater role for both secondary and primary care. It was the consensus reported here that in addition to basic sleep hygiene and psycho education, melatonin should be available within non-specialist services. The steering group supported this view as they considered melatonin to be effective in many cases, to be relatively safe, a better option than prescribing of sedative medications, and use may reduce the risk of self-treatment with cannabis or other drugs (54).

### For those treated with melatonin in childhood or adolescence, it is important to continue this treatment during the transition to adult care to ensure consistent management of sleep disorders and ADHD

A meta-analysis of randomised controlled trials evaluating the use of melatonin in children and adolescents has demonstrated its efficacy for the short-term treatment of sleep-onset insomnia, along with favourable tolerability (55). Furthermore, a separate randomised controlled trial has indicated that melatonin may be an appropriate treatment option for adults with ADHD and associated sleep disorders (25). Melatonin is the most prescribed treatment to address sleep problems in children and young people in this population (7, 32), and where effective, should be continued into adulthood with understanding that the reasons for delayed sleep onset may change as the patient ages and should be reassessed periodically.

### There is a need to develop prescribing guidelines to improve care continuity and initiation for adult patients with ADHD and sleep disorders

In accordance with NICE guidelines, it is recommended that patients undergoing treatment in paediatric healthcare services undergo a reassessment when transitioning to adult services. However, various challenges related to resources, time, structure, and organisation may result in some patients discontinuing medications, being discharged, or not being followed up during this transition phase, despite continuing to experience ADHD symptoms (56). Shared-care protocols and prescribing flowcharts should be developed to support the transition from adolescence to adulthood (7, 32).

## Strengths and limitations

This is the first study to convene key opinion leaders to discuss the optimal system of care for the management of delayed sleep onset in adults with ADHD in the UK.

A notable strength of this study is the high level of consensus observed across a large and representative cohort of 212 participants, including general practitioners, mental health service specialists, and specialists in sleep disorders from the UK. The respondents demonstrated a clear agreement with the proposed statements. However, these high levels of agreement levels may suggest a potential lack of challenge or an inclination towards consensus due to the predominance of healthcare providers practicing in England (n=187/212), resulting in limited representation from the Devolved Nations. In addition, it should be acknowledged that there are different levels of agreement/disagreement and ‘tend to agree/disagree’ responses should be considered to be weaker than ‘strongly agree/disagree’.

A limitation is our study’s inability to separate the independent and interactive effects of sleep dysregulation and ADHD on the observed outcomes, a challenge compounded by the limited research specifically addressing this comorbidity. Consequently, we acknowledge that the intertwined nature of these factors, a common issue in comorbidity research, affects the interpretation of our findings and underscores the need for future studies designed to disentangle their distinct contributions.

## Conclusions

Managing sleep disorders as a critical component of adult ADHD care is essential due to their high prevalence. Access to effective treatment options, including the appropriate use of melatonin, is necessary to improve patient outcomes. Healthcare professionals should possess a thorough understanding of ADHD-related sleep disorders and the available treatment strategies to optimise the healthcare delivery system. Initiating melatonin treatment within primary care settings may reduce the burden on specialist services while ensuring continuity of care, especially during the transition from adolescence to adulthood. Establishing clear prescribing guidelines will further enhance treatment continuity, ensuring that adult patients with ADHD and co-existing sleep disorders receive consistent, effective care throughout the healthcare system, based on the best evidence available.

## Data Availability

The raw data supporting the conclusions of this article will be made available by the authors, without undue reservation.

## Supplementary material

Supplementary Figure 1 Literature search strategy.

Supplementary Figure 2 Respondent roles.

Supplementary Figure 3 Respondent time in role.

Supplementary Figure 4 Respondents by country.

Supplementary Figure 5 Percentages of agreement level by statement.
