# Supplementary material for: The optimal system of care for the management of delayed sleep onset in adult ADHD in the UK: a modified Delphi consensus
Source: Front Psychiatry. 2025 Aug 14;16:1566390. doi: 10.3389/fpsyt.2025.1566390 (PMC12392164; doi:10.3389/fpsyt.2025.1566390)
Supplement: Supplementary Figure 1 — Literature search strategy. [file DataSheet1.docx]

**SUPPLEMENTARY INFORMATION**

**Literature review**

In March 2024, a brief literature review focused on the issue of delayed sleep onset in adults with ADHD was conducted to explore the topic and identify relevant publications using PubMed and Google Scholar platforms. A total of 53 articles published between 2017 and 2023 were identified. Various materials including articles, research papers, conference proceedings and other relevant resources were evaluated for their applicability to the current study. A general web search using free text terms was also conducted to locate any relevant literature sources not indexed within the selected databases. This additional search aimed to identify any information not indexed in the selected databases. Search terms utilised included but were not limited to “attention deficit hyperactivity disorder”, “ADHD”, and “sleep disorder”, along with related phrases to optimise the search results.

**
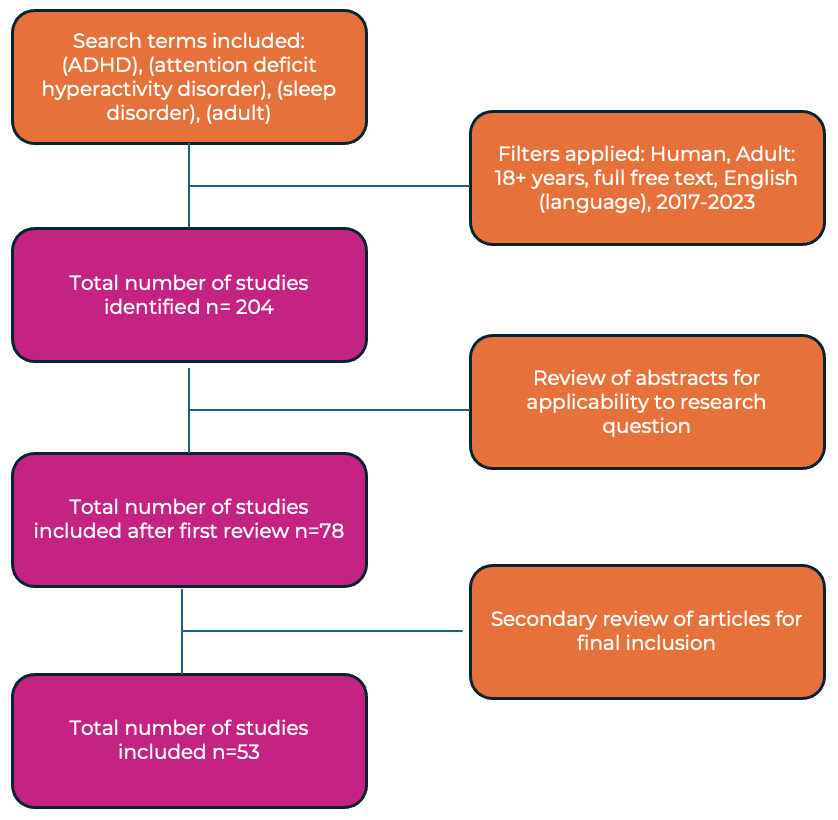
**

**Figure S1.** Literature search strategy.

**Figure S2.** Respondent roles.

**Figure S3.** Respondent time in role.

**Figure S4.** Respondents by country.

**Figure S5.** Percentages of agreement level by statement
